# Reports of Cases, Occurring in “Bellevue Hospital,” Richmond, Va.

**Published:** 1859-08

**Authors:** Thomas Pollard

**Affiliations:** One of the Physicians to the Hospital


### ARTICLE III.

*Reports of Cases, occurring in "Bellevue Hospital," Richmond, Va.*—By THOMAS POLLARD, M. D., one of the Physicians to the Hospital.

The following cases are selected, not for their novelty, or for the success of their treatment, but because it is believed, they will afford instruction. It is always a matter of interest to the medical man, to know what relations, if any, exist between the symptoms manifested during life, and the lesions found after death. Such cases truthfully recorded, with the result of a careful autopsy, aid the cause of medical advancement, more than the record of brilliant Surgical operations, or the report of diseases successfully treated by new remedies, which, oftentimes get the credit, which is alone due to nature:

CASE I. *Chronic Diarrhœa.*—Ben, slave aged 35, admitted October 1st, 1858. He is a stout man, and for a number of years has been employed in a coal-pit. Six weeks since, he was taken with diarrhœa, for which he had been unsuccessfully treated. But little emaciated, appetite good, some pain in the bowels, with three or four evacuations during the day, which were of clay color, and the consistence of mush.

Oil of turpentine and laudanum, sub. nit., bismuth, sulphate of copper and opium, sulphate of morphia with sulphate of copper, extract of gentian and strychnia, minute doses of mercury, nitrate of silver and extract of hyosciamus, nitrate of iron, Hope's mixture, acetate of lead and opium, tannin and blisters, were all used without permanent good effect. Cod-liver oil and stimulants were also used. The passages were reduced at times to one in the twenty-four hours, but they maintained, generally, their white, unhealthy color, with copious quantity. For 2 months he remained up, and walked about the house during the day. For one month previous to his death, emaciation was rapid, and he kept his bed most of the time. Tympanitis supervened, the tongue became more glazed, and was ulcerated, and to this succeeded involuntary evacuations, great prostration and death, which occurred January 11th, 1859. P. M., 12 hours after death, the surface of the colon was thickly studded with ulcers its entire length. The entire intestinal tube was more or less congested, and all of the mesenteric glands were much enlarged. Kidneys enlarged and fatty. The liver presented also some fatty degeneration. Lungs healthy.

There is a doubt in this case whether the enlargement of the mesenteric glands was primary or secondary. The diagnosis before death was "Tubercular disease of the mesenteric glands," and the probability is that the disease of the glands, was the *first* chain in the link of morbid actions.

Case 2. *Pneumonia with Endocarditis.*—Armstead, slave, aged 45, admitted Nov. 11, '58. Auscultation and percussion revealed pneumonia in the left lung, in the second stage. There was dullness on percussion, with bronchial breathing, and bronchophony. He complained of severe pain in the region of the heart, with dyspnea, cough, and some viscid expectoration. The action of the heart was labored, and the signs pointed to some endocarditis. He reports that he had never been sick before this attack, and that he had now been sick for 3 weeks. Nov. 12. ℞.—Cups to 6 ozs., over the region of the heart; submur. hyd., grs. 5; pulv. dov., grs. 10; opium, 1 gr., to be given immediately, to be followed by submur. hyd., grs. 2; pulv. dov., grs. 3, every 3 hours. Nov. 13.—Cupping and medicine have given considerable relief—the pain and dyspnea being much relieved. Pulse 95 per minute, full and strong. ℞.—Blister, 8 x 10, to the left side; veratrum viride, 7 gtt. every 4 hours. Nov. 14.—Feels better; pulse reduced 20 beats in the minute; cough troublesome; suspend the veratrum viride. At 11 o'clock, p. m., was quiet; had but little pain, and cough less troublesome; been taking "Brown mixture" for the cough, during the day. Nov. 15.—Was found dead.

Autopsy 36 hours after death.—Superior ⅓ of the left lung solidified, and the other portion congested; heart hypertrophied (especially the left ventricle), weighing 11½ ounces; the semi-lunar valves appeared healthy; the others, more or less diseased; mitral valve much inflamed, and thickened; in the left cavities, and extending into the aorta, and its branches, were several masses of coagulated fibrine, one piece being 25 inches in length. The aggregate weight of the pieces being 14 ounces.

It is an interesting enquiry to know what agency these coagula had in producing death. The amount of lung diseased was not sufficient to cause sudden death; the right lung being sound. There can be no reasonable doubt, but what such coagula may arrest the circulation of the blood, and the action of the heart. And recent observations have proved, that portions of these fibrinous deposits, by detaching themselves, may be carried by the current of the blood into the circulation, and block up arteries of considerable size, and produce in the organs the consequences of defective supply of blood. Softening of the brain, and hemiplegia are believed to have been thus caused.

Dr. Gerhard believes these coagula to be more frequently the cause, than the effect of endocarditis, and ascribes their origin to the highly fibrinous condition of blood found in inflammatory diseases.

CASE 3. *Epilepsy.*—Lewis, aged 19, admitted Nov. 10, '58. He is idiotic, and can give no account of himself. His master says he has been "fitty" nearly all of his life, but up to a few months, worked at his usual employment. For some weeks he has had almost daily attacks of the disease. For two days after his admission, he had several attacks during the day and night, which were more or less severe. Generally the *right* side was convulsed, while the *left* was perfectly quiet; but sometimes the reverse of this took place. 13th Nov.—The paroxysms became more frequent, but less severe; the intervals being scarcely more than five minutes. Coma came on, and death occurred on the 15th. The treatment, enemas of turpentine and assafœtida, cold, to the head, and blisters did no good. P. M.

appearances.—The dura mater adhered very tightly to the calvarium, through almost its whole extent. There was extensive effusion into the *right* lateral ventricle, and none in the *left*. The medulla oblongata and *left* side of the cerebrum were hardened. In the *left* cerebrum, about ¼ of an inch from the exterior, was found a cavity 2 by 1½ inches. The membrane lining this cavity was thin, and considerably inflamed.

This was a case of apoplexy, following repeated attacks of epilepsy, which had by degrees led on to congestion, and rupture of a blood vessel, or effusion. The convulsive action on the right side corresponded to the lesion found in the left cerebrum.

Case 4. *Pleuropneumonia followed with Empyema and Anasarca.*—Richard, aged 14, admitted May 11, '59. Anasarca throughout the lower extremities. Face, hands and chest puffy, with some effusion. The ear applied to the chest detects a great amount of effusion into the bronchia, an unusual degree of mucous rales being audible. The upper part of the chest on percussion, not clear—the lower part very dull. Dyspnea urgent, pulse 100, and weak. The diagnosis made, was "œdema of the lungs," with fluid in the pleural cavity of both sides.

The chest was blistered, and submur. hyd., grs. 2; pulv. dov., grs. 2; syrup squills, 1 dr., and spts. nitre, 1 dr., directed every 2 hours. Whisky toddy and juniper berry tea every 2 hours. In spite of medicine and stimulants, the patient's system went down. May 12.—Died. P. M. appearances.—Both pleural cavities half filled with pus, and with flakes of coagulable lymph unorganized, as large as the hand, floating in the pus. The whole of the lungs more or less œdematous; more perceptible about the edges and free surfaces. The bronchial tubes filled with mucous and serum.

The history of the case obtained from an intellectual physician, was, that the boy had had double pneumonia, with pleurisy. He was considered convalescent, and at one time walked about. In a week or so, later, the symptoms under which he was laboring, when brought to the hospital, commenced. This case teaches the importance of watching the convalescence from serious disease. Could the existence of pus been certainly known, here would have been a justifiable case for "Paracentesis Thoracis."

Case 5. *Cavity of the Lung, probably the result of Pneumonia.*—Ben, aged 25, admitted May 3, '59. He had been laboring under "Pertussis," from which he was at one time supposed to have nearly recovered. At the time of his admission, he had much dyspnea, pulse 100, with respiration, 60 in the minute. The physical signs were those of quite extensive bronchitis.

℞.—Submur. hyd., grs. 3; pulv. dov., grs. 2, in 1 dr. syrup senega, every 2 hours. Beef tea, with weak toddy. May 5.—Much the same; give the medicine every 6 hours. May 6.—Continue treatment. May 7.—Cough more troublesome, and spasmodic. ℞.—Cochineal, grs. 5; hive syrup, gtts. 25; carbonate potass., grs. 5, every 3 hours. Stop the other medicine. May 15.---Symptoms growing more unfavorable. Deep blister over the chest, to be dressed with "mercurial ointment;" stimulants more freely; continue medicine. Patient grew worse, and died on the 19th May. P. M. appearances.—Bronchitis existed on each side quite extensively. In the left lung, at the union of the superior and lower lobes, not far from the surface, a *cavity* was found large enough to contain ½ pint. This cavity contained pus, and was lined by a pus secreting membrane, apparently of long existence. for there were but little remains of congestion of the part, or injection of the vessels. This cavity had no connexion with the bronchia, and was evidently the result of pre-existing *pneumonia.* In connexion with this, the man stated, he had had an attack of some kind of sickness, several years ago, and had had a cough ever since. The position of the cavity, and the absence of any tubercles, in either lung, make it all but certain that the cavity was the result of abscess of pneumonia.

A medico-legal question arises out of this case. The man had been purchased about 3 months; the hooping-cough came on subsequently to this, while he was in a jail in the city; a question of soundness was raised at the time of purchase; we gave it as our opinion, that the cavity had, in all probability, existed at the time of purchase, and perhaps, for a year or two. The hooping cough and bronchitis seemed to be the immediate cause of death. Though it was probable, that if this had not been added to a previously unsound lung, that the patient might have survived.

CASE 6. *Typhoid Fever.*—Alpheus, admitted June 17, aged 25, stated he had been feeling badly for several weeks, but had kept at his work until the last few days; was sitting up when brought to the hospital; was having 2, 3 or 4 passages a day; pulse only 80; tongue slightly furred; pulse rather full and labored. From the character of the pulse, doubted whether this was a case of diarrhœa or typhoid fever. ℞.—Oil of turpentine, 10 gtts., and laudanum, 8 gtts., every 4 hours. Diet: Milk, cold bread, and chicken broth. June 18.—Found a considerable disposition to somnolency. Passages of thin, fecal, yellow character, such as are usually found in typhoid fever, came to the conclusion, that this was certainly a case of typhoid fever. Add 5 grs. mur. ammonia, to the laudanum and turpentine, and gave every 6 hours. Pulse rather active; skin hot. June 19.—Found the quantity of urine secreted small in quantity, very dark colored, and voided with difficulty. Continue treatment, with addition of spts. of nitre, ʒi. to the other medicines. June 20.—Found no urine had been passed for more than 24 hours. Catheter introduced, and about a quart of very dark urine drawn off. This left, on standing, a dark mucous deposit, with some appearance of a dark powder at the bottom. Thought this might be blood, but on testing it, found it not to be. No albumen in the urine, with acid reaction. Somnolency continues. Passages more consistent. Some slight wandering during his sleep. June 25.—Patient remains in nearly the same condition, except, that somnolency has increased, passages less frequent, tongue not looking very unnatural. June 26.—Some dilatation of the pupils. Laudanum was discontinued. Blister to nape of the neck and back part of the head. Urine has gotten in better condition, and there has been no retention of it since the 20th. Passages more natural. June 29.—Somnolency about the same—can be roused without much difficulty, and talks rationally, but with hesitation, when roused. Takes some nourishment; pupils still dilated. Continue same medicines. June 30.—Had a decided spasm—the whole system being convulsed—and is, at 12 o'clock, M., in a profound coma. Scalp to be shaved, and a blister applied over the whole surface of the scalp. July 1.—Died about 4, P. M., having had repeated spasms, but none for the last 12 hours.

Autopsy 14 hours after death.—On opening the brain, found some coagulable lymph on the surface of the meninges covering the cerebrum, and considerable injection of the vessels. No effusion in the ventricles. Much effusion about the base of the brain, with injection of the vessels which accounts for the convulsive action. On opening the small intestines, 2 very large, dark, sloughy looking ulcers were found in the ileum, near the ileo-colic valve, and the solitary glands were much enlarged. The whole of the small intestinal mucous membrane very much injected. The stomach inflamed, and the mucous membrane dark, inflamed, thickened, and softened. The kidneys were not examined, as they should have been. Large intestines healthy, containing healthy looking fecal matter. It was afterwards ascertained, that the patient had been for some time a drinking character, and this probably, accounts for the unusual amount of inflammatory action, found in the stomach and small intestines. Connected with this, was an obstinate vomiting, not mentioned, which prevented the patient from retaining food and medicine. And this case is another of the frequent evidences of some lesion of nutrition, which so often seems connected with the causation of typhoid fever.

We believe the majority of cases of the fever, occur in persons whose system has been weakened in some way, by bad food, bad air, late hours, and watching, and excessive mental and bodily fatigue. The stomach and bowels become weakened, and predisposed to take on the peculiar inflammation found in the "elliptical patches" of the ileum. The peculiar typhoid fever poison existing in the air, is then the exciting cause to this predisposed condition. The almost healthy condition of the tongue is another noticeable thing in this case, notwithstanding the very unhealthy state of the stomach. It is remarked by some, that the tongue, bears no fixed relation to the condition of the mucous membrane of the intestines and stomach, in typhoid fever, and this case supports this view.

